# A *Salmonella* Typhimurium outbreak linked to Vietnamese bread rolls in South Western Sydney, Australia, 2015

**DOI:** 10.5365/WPSAR.2016.7.2.007

**Published:** 2017-06-21

**Authors:** Meena Chandra, Heidi Lord, Stephanie Fletcher-Lartey, Kate Alexander, Nilva Egana, Stephen Conaty

**Affiliations:** a Public Health Unit, South Western Sydney Local Health District, Sydney, Australia.

## Abstract

**Introduction:**

In September 2015, the South Western Sydney (SWS) Public Health Unit was notified of a cluster of *Salmonella* Typhimurium (STm) cases with a common multiple-locus variable-number tandem repeats analysis (MLVA) pattern. An investigation was conducted to identify a source and contain the outbreak.

**Methods:**

The cluster was initially identified through routine geographic information system cluster scanning applied to the New South Wales Notifiable Conditions Management System. Additional cases were identified through a complaint to local council about a bakery. The bakery was inspected and 48 environmental and food swabs were collected for analysis.

**Results:**

A total of 26 suspected cases were identified, of which 14 were interviewed. STm MLVA type 3–16–9-11–523 was identified in 19 of 26 case stool specimens. Most cases (12/14) consumed bread rolls containing pork or chicken with chicken liver pâté and raw egg mayonnaise filling. Five cases identified a common bakery exposure. Environmental and food samples from the bakery isolated STm with an identical MLVA pattern.

**Discussion:**

An STm cluster in SWS was investigated and found to be linked to Vietnamese bread rolls containing pork or chicken with chicken liver pâté and raw egg mayonnaise filling. Confirmation of a distinct MLVA pattern among STm isolates from clinical, food and environmental samples provided evidence to establish an epidemiological link between the cases and the implicated premises and informed public health action to contain the outbreak.

## Introduction

Approximately 4.1 million cases of domestically acquired foodborne gastroenteritis occur in Australia annually. *Salmonella* is a frequently implicated organism and is responsible for the majority of hospitalizations and deaths attributable to foodborne infections. *Salmonella* Typhimurium (STm) is the most common serovar in Australia. (*1*)

In New South Wales (NSW) salmonellosis is a notifiable condition under the Public Health Act 2010. Laboratories are required to report positive culture results of *Salmonella* species to NSW Health. In NSW, *Salmonella* isolates are referred to Pathology West – Institute for Clinical Pathology and Medical Research, the state reference laboratory, for further characterization, including serotyping and DNA sequence-based subtyping with multiple-locus variable-number tandem repeats analysis (MLVA). (*2*) The data are entered into the NSW Notifiable Conditions Information Management Systems (NCIMS) managed by NSW Health. NSW Health routinely tracks *Salmonella* using SaTScan v9.4.2, a geographic information system (GIS) software programme, to identify spatiotemporal clusters of STm that have been notified through NCIMS. SaTScan can detect spatial patterns and disease clusters before obtaining MLVA typing results, which can take up to two weeks to be completed.

In September 2015, the Communicable Diseases Branch of NSW Health alerted the South Western Sydney Public Health Unit to a geographical cluster of seven cases of STm infection, in residents of South Western Sydney (SWS). The cases symptom onsets appeared to be clustered in time from 2 to 14 September 2015, lived in close proximity to each other and had South-East or East Asian surnames. Six of the seven cases initially tested had a common MLVA pattern (3–16–9-11–523), suggesting an epidemiological link. An investigation was then undertaken to confirm cases, characterize and identify a common source to control the outbreak and prevent future outbreaks.

## Methods

### Epidemiological investigation

To investigate this STm cluster, the following case definitions were developed:

1. A suspected case was defined as a resident of SWS with onset of symptoms (vomiting, diarrhoea, and/or abdominal pain) in the first two weeks of September 2015 with an epidemiological link (similar exposure, similar food product, relative or carer of case) to the cluster.
2. A confirmed case was a suspected case with laboratory-confirmed evidence of STm with MLVA pattern 3–16–9-11–523.

All suspected and confirmed cases were contacted via telephone and interviewed after consent was obtained. Each case was interviewed using a nationally validated standardized *Salmonella* hypothesis-generating questionnaire. Information about clinical presentation and onset date, hospital admission and treatment, and contact and environmental exposures was obtained. Specifically, information on home food purchases, eating outside the home, special diets and open-ended questions on food consumed was obtained for the seven days before symptom onset. A further section detailed a range of specific high-risk foods that may have been consumed during the priority period. Data were entered into Microsoft Excel 2010 for analysis.

### Environmental investigation

In response to the complaint made about the bakery, the New South Wales Food Authority (NSWFA) was informed and an inspection of the premises carried out on 25 September 2015. Food and environmental swab samples (*n* = 48) were collected from around the bakery and sent for laboratory testing.

## Results

### Epidemiological investigation

**Fig. 1:**
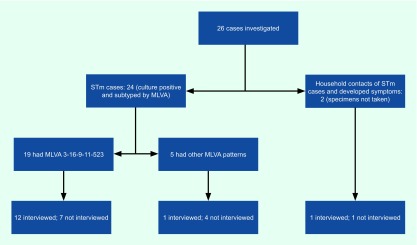
**Recruitment of cases**

A total of 26 suspected STm cases were identified in SWS between 1 and 15 of September 2015; 24 had STm isolated from stool specimens and were entered into NCIMS. The remaining two were household contacts of two cases that had STm isolates who made a complaint to the SWS local council. The household contacts had similar food exposures to the notified cases and developed symptoms during the same period; however, stool specimens were not obtained from them. MLVA patterns were available for the 24 cases from whom STm were isolated. Of these 24 cases, 19 had a common MLVA pattern 3–16–9-11–523, and the other five had other MLVA patterns (**Fig. 1**).

Twelve of the 19 cases with the MLVA pattern 3–16–9-11–523 were interviewed. In addition, one of the five cases with a different MLVA pattern was interviewed before the MLVA results were available, and one case linked to the cluster but whose specimen was not taken was also interviewed (**Fig. 1**).

**Table 1** describes the demographic and exposure profile of the 14 interviewed cases. The cases ranged in age from 1 to 77 years old with 43% between 1 to 16 years old. There were more females (57%) than males (43%). Of the nine (64%) cases interviewed, seven presented to the hospital emergency department (seven required admission and two were treated in the emergency department). The majority of cases reported eating pork rolls (64%).

**Table 1:** Demographic and exposure characteristics for interviewed STm cases, South Western Sydney, Australia, 2015

Characteristic	n	(%)*
Gender	
Female	8	57
Male	6	43
Age group (years)	
1–16	6	43
17–30	2	14
31–40	3	21
40+	3	21
Hospital presentation	
Yes	9	64
No	5	36
Food exposure	
Pork roll	9	64
Chicken roll	3	21
No roll	2	14

* Percentages are rounded to nearest whole number and may not add up to 100%.

Of the 12 confirmed cases interviewed, 11 consumed a Vietnamese roll with mayonnaise and pâté (eight containing pork and three containing chicken) from a bakery, and the other case consumed a beef dish in a restaurant. Among the 12 interviewed, four purchased the roll from a specific bakery within 24 hours before symptom onset, while six cases purchased a Vietnamese roll in the same vicinity/postcode but could not recall the street or bakery name. The remaining two cases did not recall eating a Vietnamese bread roll in the area of interest. No other common food exposure was identified.

### Environmental investigation

Findings from the inspection found the bakery sells up to 300 Vietnamese bread rolls containing raw egg mayonnaise/butter and pâté mix daily.

Samples from the chicken liver pâté mix taken from the storage fridge, raw meat (pork), pâté blender and blade and a shoe swab from the food preparation area had identical MLVA patterns to the cluster.

### Control measures

The NSWFA issued a prohibition order on the sale of all bread rolls from the implicated bakery. Follow-up environmental testing was all negative, and the prohibition order was removed once the NSWFA was satisfied that food safety knowledge and practices had improved. The bakery has been banned from using raw egg mayonnaise or chicken liver pâté as they were not able to demonstrate sufficient expertise in the safe preparation and storage of these items.

## Discussion

The application of spatiotemporal cluster scanning and MLVA typing enabled the detection of an outbreak of STm in NSW that facilitated a multiagency intervention to prevent further spread of the infection. The outbreak was linked to the consumption of Vietnamese bread rolls (containing pork or chicken with chicken liver pâté and raw egg mayonnaise filling) with an epidemiological and microbiological link to a common source. Molecular typing identified the same MLVA pattern found in several cases in the pâté and pork sampled from the bakery, confirming these items as the likely sources of infection in cases that were linked to the bakery. In previous reports, eggs, pork, chicken and salad rolls have been implicated in large outbreaks. (*1*-*5*)

The detection of STm from foods and surfaces around the implicated bakery suggests substandard food handling and general hygiene practice. A survey conducted by Food Standards Australia New Zealand in 2007 found that food handling in bakeries was less compliant than in other types of businesses. (*6*) Other factors contributing to *Salmonella* outbreaks include inadequate storage and refrigeration, the use of expired eggs, mixing of old and new batches of food items and poor general cleaning practices. (*4*)

Foodborne disease costs Australia 1.2 billion Australian dollars (Aus$) each year, largely due to hospital presentations, losses in productivity from days off work or caring for affected family members. (*1*, *3*) In 2000, the Australian Government established OzFoodNet as a joint initiative with Australia’s state and territory health authorities to improve national surveillance of foodborne outbreaks, identify ways to minimize foodborne illness and to further understand the causes of foodborne illnesses. In 2008, a study conducted by OzFoodNet found that the costs averted from successful outbreak investigations was between Aus$ 85 000 and Aus$ 1.3 million due to early identification and removal of contaminated food from the food supply chain. (*1*) Early identification and removal of these foods in this outbreak was critical in minimizing the costs associated with further cases.

This study has several limitations. Not all cases with a matching MLVA were interviewed; hence, it is unclear what the source of their infection was. Additionally, due to time constraints and resources, controls were not recruited for the study, which could have further strengthened the evidence against the implicated food items and bakery. However, the distinct MLVA pattern among *Salmonella* isolates from clinical, food and environmental samples provided strong evidence to establish an epidemiological link between the cases and the implicated premises. Lastly, sufficient data were unavailable to examine egg mayonnaise as a potential source.

The application of GIS to routine surveillance enabled the detection of geospatial clustering of STm cases with an identical MLVA pattern in NSW in September 2015. In-depth investigation established an epidemiological link between several cases and food and environmental samples taken from an implicated bakery. While it was not possible to link all cases with the same MLVA pattern to the bakery, the evidence enabled local health officials to carry out enforcement actions that led to the business being banned from preparing their own chicken liver pâté and raw egg mayonnaise, restricting the spread of STm within the community.
